# Exploring Iguape Virus—A Lesser-Known Orthoflavivirus

**DOI:** 10.3390/v16060960

**Published:** 2024-06-14

**Authors:** Marielena V. Saivish, Maurício L. Nogueira, Shannan L. Rossi, Nikos Vasilakis

**Affiliations:** 1 Laboratórios de Pesquisas em Virologia, Departamento de Doenças Dermatológicas, Infecciosas e Parasitárias, Faculdade de Medicina de São José do Rio Preto, São José do Rio Preto 15090-000, SP, Brazil; marielenasaivish@gmail.com (M.V.S.); mauricio.nogueira@edu.famerp.br (M.L.N.); 2 Brazilian Biosciences National Laboratory, Centro Nacional de Pesquisa em Energia e Materiais (CNPEM), Campinas 13083-100, SP, Brazil; 3 Department of Pathology, University of Texas Medical Branch, Galveston, TX 77555-0609, USA; slrossi@utmb.edu; 4 Center for Vector-Borne and Zoonotic Diseases, University of Texas Medical Branch, Galveston, TX 77555-0609, USA; 5 Institute for Human Infection and Immunity, University of Texas Medical Branch, Galveston, TX 77555-0610, USA

**Keywords:** *orthoflavivirus*, mosquito-borne virus, arbovirus, transmission cycles

## Abstract

Brazil has earned the moniker “arbovirus hotspot”, providing an ideal breeding ground for a multitude of arboviruses thriving in various zoonotic and urban cycles. As the planet warms and vectors expand their habitat range, a nuanced understanding of lesser-known arboviruses and the factors that could drive their emergence becomes imperative. Among these viruses is the Iguape virus (IGUV), a member of the *Orthoflavivirus aroaense* species, which was first isolated in 1979 from a sentinel mouse in the municipality of Iguape, within the Vale do Ribeira region of São Paulo State. While evidence suggests that IGUV circulates among birds, wild rodents, marsupials, bats, and domestic birds, there is no information available on its pathogenesis in both humans and animals. The existing literature on IGUV spans decades, is outdated, and is often challenging to access. In this review, we have curated information from the known literature, clarifying its elusive nature and investigating the factors that may influence its emergence. As an *orthoflavivirus*, IGUV poses a potential threat, which demands our attention and vigilance, considering the serious outbreaks that the Zika virus, another neglected orthoflavivirus, has unleashed in the recent past.

## 1. Introduction

In recent years, neglected arboviruses have taken center stage as severe outbreaks in various countries [1,2,3,4,5,6,7,8,9,10,11,12,13] have strained healthcare systems and incurred enormous socioeconomic costs [14,15,16]. The global spread of arboviral infections, propelled by expanding mosquito habitats due to heightened trade, uncontrolled urbanization, and climate change, has increased awareness among public health, research, and policy stakeholders [17,18,19,20]. Iguape virus (IGUV), a single-stranded positive-sense RNA virus within the *Orthoflavivirus aroaense* species (*Flaviviridae,*/*Orthoflavivirus*) [21], was initially isolated in 1979 from sentinel mice in São Paulo State, Brazil, following a Rocio virus (ROCV) outbreak [22]. 

Brazil, renowned for its sprawling ecotypes and biodiversity, has long been considered an “arbovirus hotspot” for fostering ideal conditions for numerous arboviruses in diverse zoonotic and urban transmission cycles [4,12,23,24,25,26,27,28,29,30,31,32,33,34,35]. For IGUV, a potential emergent threat, to date, there is limited information available on its transmission cycles and pathogenicity in both animals and humans. Additionally, the burden of IGUV infections may be significantly underestimated, given the lack of accurate diagnostics and its omission from laboratory screenings. Similar to the trajectories of Zika (ZIKV) and chikungunya (CHIKV) [36] viruses, IGUV infections may evolve into a major health concern. Notably, the existing literature on IGUV spans decades, is outdated, and is often a challenge to access. Amidst the warming planet and expanding vector habitats [19,37,38,39,40], we delve into the known IGUV literature to review the information that is known, identify gaps, and suggest comprehensive studies on the biological aspects, potential vectors, and transmission dynamics that are urgently needed.

## 2. Discovery, Classification, and Taxonomy

Since 1961, the Section of Arthropod-Transmitted Viruses (S.A.T.V.) at the Adolfo Lutz Institute, a key institution affiliated with the Department of Health of the State of São Paulo, has been conducting ongoing studies in ecology and epidemiology focused on arbovirus infections in the Atlantic rainforest regions of this state. These investigations involved collecting vectors and blood samples from wildlife in the area and were instrumental in the detection and response to the largest recorded encephalitis outbreak in the country in 1975 caused by ROCV (reviewed in [25]). This outbreak affected over 1000 people in the Vale do Ribeira region, with a 10% fatality rate, and 20% were affected with long-term sequelae [25,26]. The response to this outbreak included sustainable surveillance efforts, with the capture of animals and vectors, as well as the use of sentinel animals for the early identification and characterization of new arboviruses. 

In January 1979, a new virus, initially designated as SPAn 71686 and later renamed Iguape virus (IGUV), was isolated from sentinel mice in the Atlantic rainforest region within the municipality of Iguape, state of São Paulo. The mice were brought into the laboratory for further observation, where visible signs of infection were noted, including tremors, paralysis, and lethargy. Mice were euthanized, their brains were collected, and filtered brain homogenates were intracerebrally inoculated into suckling mice. The virus was subsequently identified using the complement fixation test [22].

Currently, there is limited available information on the molecular characteristics of IGUV. Ultrastructural observations of mouse brain tissue collected 73 h post-inoculation revealed viral particles predominantly in the cytoplasm of infected cells, as well as in extracellular spaces, with an approximate size of 41 nm [22]. The virus is currently classified as a member of the genus *Orthoflavivirus*, which encompasses over 70 virus species and is within the Aroa antigenic complex [21,41]. IGUV demonstrated pathogenicity in several laboratory animals where high viral titers were observed in the brains of Swiss mice and suckling hamsters, which developed fatal encephalitis six days post-inoculation via the intracerebral route. However, young adult hamsters (6–8 weeks old) inoculated intraperitoneally developed an encephalitic illness from which they eventually recovered [22].

## 3. Experimental Studies on Ecology and Transmission Cycles

Following the IGUV discovery, serological surveys were conducted on animals in the region to gain a better understanding of potential viral reservoirs and affected animals in an attempt to trace a possible transmission cycle [22,42]. The studies focused on animals in the Vale do Ribeira and Vale do Rio Iguape regions because of their rich fauna. Serological surveys were conducted on bird samples collected between 1989 and 1990, showing monotypic response to IGUV in 50 birds from the following 16 families: *Columbidae*, *Furnaridae*, *Formicariidae*, *Conopophagidae*, *Piiridae*, *Tyrannidae*, *Hirundinidae*, *Troglodytidae*, *Turdidae*, *Motacillidae*, *Plodeidae*, *Vireonidae*, *Icteridae*, *Parulidae*, *Thraupidae*, and *Fringillidae* [42] (Table 1). Many of the identified birds were resident–migratory species, notably *Myiarchus swainsoni* (*Tyrannidae/Myiarchus*) [42]. This passerine bird, commonly known as Swainson’s flycatcher or *Swainson’s Myiarchus*, is renowned for its extensive migration patterns, suggesting a possible role in IGUV’s long-distance dispersion. During the breeding season, they migrate from South America to northern regions of the continent, including parts of Central America [43]. *Vireo olivaceus* (*Vireonida,/Vireo*), on the other hand, is a small songbird commonly known as the red-eyed vireo, which is primarily found in North and Central America and encompasses a vast geographical range. During the breeding season, they inhabit deciduous forests, but they undergo extensive migrations, moving to wintering grounds in Central and South America [44]. Given that many arboviruses (e.g., West Nile (WNV) [45,46], Ilheus (ILHV) [47], Saint Louis encephalitis (SLEV) [48], ROCV [26,42] and others) infect wild birds and can be amplified at high levels of viremia that make birds infectious to various vector species, it has been suggested that the migratory bird movements could represent a crucial mechanism for the dispersal of these viruses on a local, continental, and intercontinental scale [49,50].

Between 1989 and 1992, Coimbra and colleagues performed serological studies based on the hemagglutination inhibition (HI) test on wild rodents, marsupials, teal, ducks, and chickens, and showed the presence of flavivirus antibodies (Table 1) [22], suggesting their possible role in the transmission cycle. Critically, though, they demonstrated that wild birds had a monotypic response to IGUV, strongly suggesting a key role in the transmission of the virus. Notably, the tested wild bird samples (*n* = 973) were representative of 33 species belonging to 29 genera and representing 17 families, showing a 9.89% (46/465), 18.90% (40/212) and 19.50% (58/296) positivity rate in 1990–1992, respectively [22]. A similar monotypic response was shown in chickens and ducks, raising a notion that may play a role as bridge hosts of IGUV transmission in urban settings, given their proximity to humans as they are commonly raised in urban and rural environments, often sharing spaces close to human residences. 

The highly primatophilic *Anopheles (An.) cruzii* mosquitoes collected in 1994 from the city of Juquitiba, located in the Vale do Ribeira region about 80 miles from the city of Iguape [51], provide, to date, the only record of IGUV detection and isolation from naturally infected mosquitoes. Notably, *An. cruzii* mosquitoes are considered the primary vectors of transmission for humans and simian malaria in the Brazilian regions covered by the Atlantic Rainforest [55,56,57]; however, *Anopheles* spp. are known to be competent vectors of transmission for the o’nyong nyong virus (ONNV), an arbovirus endemic in East Africa [58] and possibly Cacipacore virus (CPCV), a zoonotic arbovirus endemic to Brazil [29,59]. Their high abundance is predominantly in the hills of the Vale do Ribeira, which has intense deforestation and land use changes which may not only have created favorable ecological and microclimate conditions (e.g., natural breeding sites) that may favor the distribution and relative abundance of certain vectors of transmission but also the pathogens they transmit, partly resulting from their opportunistic behavior and feeding habits [56,60,61]. Lastly, additional information based on experimental vector competence is still necessary to determine the role of *An. cruzii* in the transmission cycle of IGUV.

**Figure 1:**
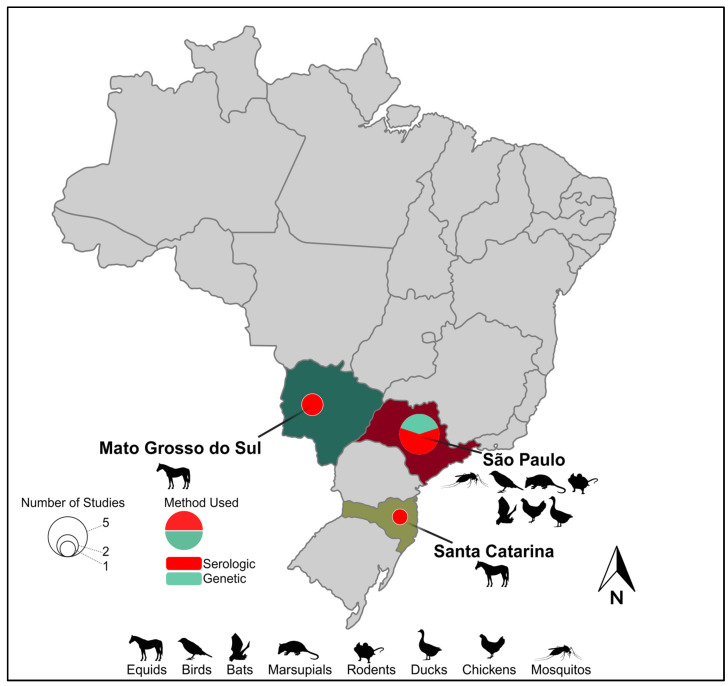
Geographic range and epidemiological landscape of Iguape virus. Brazilian states with evidence of IGUV circulation are named. Hosts from which IGUV and/or antibodies have been identified within a given Brazilian state are indicated by a representative graphic(s). Pie charts within a given state indicate the number of studies identifying IGUV by size and the method of their identification by color.

The proximity of horses to human populations is also a concern regarding the dissemination of diseases [62]. Horses are often found in both urban and rural areas bordering forested areas, where they share spaces close to humans for recreational activities, sports, or work. This proximity creates a potential interface for the transmission of arboviruses (e.g., IGUV) between horses and humans through the bites of generalist mosquitoes. Several serosurveys in various regions of Brazil have been performed in the first decade of the 21st century aimed at determining their role in IGUV transmission and have detected their presence in horses sampled at the states of São Paulo State [52,53], Mato Grosso do Sul [53,54], and Santa Catarina [53], demonstrating the circulation of this virus in the central and southern region of Brazil (Figure 1). Beyond the unknown impact of IGUV infection on the health of horses, experimental studies are urgently needed to assess whether horses could serve as reservoirs and/or amplification hosts, thus expanding IGUV’s host range and its potential to seed urban outbreaks.

**Figure 2:**
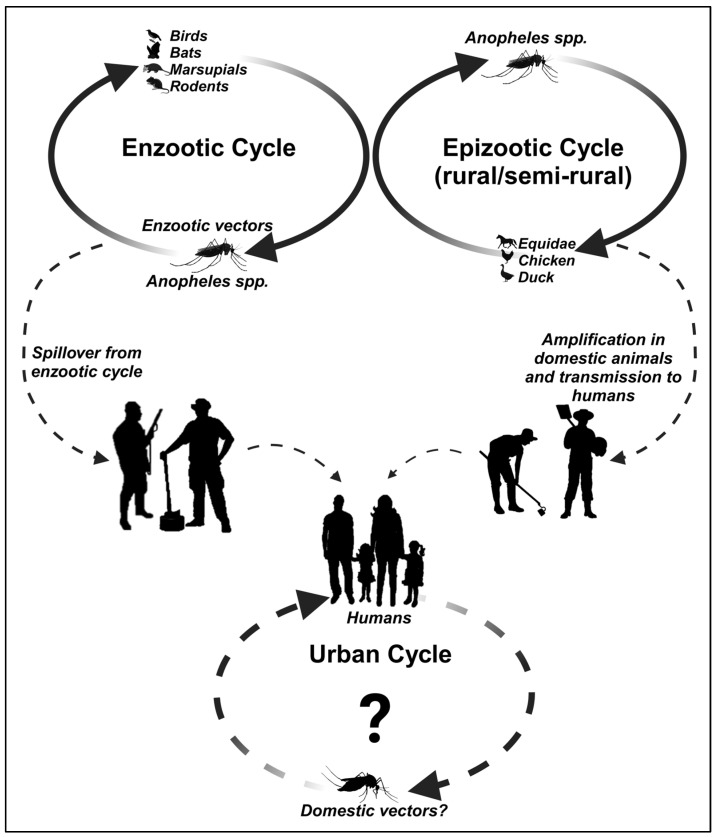
The possible transmission cycles of IGUV.

To date, there are no known reports in the literature of IGUV infections in humans nor prevalent data in serological surveys of humans. A possible IGUV transmission cycle is proposed in Figure 2 based on the currently available information in the literature and its potential role in human infections, given that the absence of any evidence is purely speculative. Moreover, it is important to note that despite the detection of monotypic and heterotypic antibodies in serological surveys that identified various vertebrates as potential vertebrate hosts (other than sentinel mice) of IGUV transmission, as reviewed above, no acute infections in animals or humans that could incriminate IGUV as a pathogenic agent have been observed so far.

## 4. Diagnosis, Treatment, and Prevention

As mentioned above, there is no available information on the range of clinical manifestations of IGUV infection in humans. As an exceptionally poorly understood virus, there are no commercial diagnostic tests available as IGUV is not routinely included in any panels of laboratory diagnostic protocols of public health centers in Brazil. Only a few research centers in the country possess the infrastructure and adequate resources needed for its identification, contributing to our lack of understanding of IGUV circulation and, consequently, the actual impact IGUV may have on veterinary and human health across Brazil and beyond. Diagnostic tests mentioned in the literature are in-house research laboratory-developed tests such as the hemagglutination inhibition test (HI) [22,42,52,53,63] and the plaque reduction neutralization test (PRNT) [54,63], used mainly for serological testing. Molecular testing involves RT-qPCR [64] in addition to viral isolation [22,63]. Therefore, due to the lack of infrastructure and limited resources for accurate IGUV identification, an IGUV outbreak could go unnoticed and likely be attributed to other causes, given that Brazil is endemic for various orthoflaviviruses (e.g., SLEV, ROCV, ILHV, CPCV, ZIKV) and other tropical diseases (e.g., malaria) that present with a similar range of symptoms. Therefore, improving diagnostic capabilities is crucial, given the notorious cross-reactivity among orthoflaviviruses [41]. Developing specific serological tests that can accurately distinguish IGUV from other orthoflavivirus infections, while challenging, is essential for the rapid and accurate detection of IGUV in low-resource settings.

There is no licensed vaccine or antiviral treatment for IGUV infections, and given the absence of any documented human infections, the development of an IGUV vaccine candidate may be challenging and unrealistic. The detection of antibodies in animal serological surveys [22,42], while indicating the circulation of the virus in various vertebrate species, has not been accompanied by reports of any veterinary disease manifestations, thus further obfuscating its disease burden in vertebrate animals. On the other hand, IGUV may undergo stochastic mutations over time, leading to vector host range changes, changes in its virulence, or ability to infect humans, leading to its rapid emergence and dissemination across the globe; these are events that have, over the last two decades, been experienced with the emergence from obscurity and global distribution of ZIKV [65,66], CHIKV [67] and SARS-CoV-2 [68,69]. Recent efforts by world bodies (e.g., The World Health Organization (WHO) [70] or the Coalition for Epidemic Preparedness Innovations (CEPI) [71] have ramped up efforts to better predict and respond to sudden attacks by unknown pathogens—also referred to as Disease X—by investing in new methods for the rapid development and deployment of effective countermeasures, such as vaccines or antivirals, as proactive strategies to respond to potential future outbreaks.

All currently available evidence suggests that IGUV may be primarily confined to regions within Brazilian biomes. However, the possibility that IGUV circulates elsewhere in Central and South America and the Caribbean cannot be ruled out. Despite this apparent restriction, occasional spillovers, emergence, and the global spread of the virus cannot be ruled out, as witnessed with WNV [72,73], CHIKV [74,75,76,77,78], and ZIKV [6,79,80,81,82,83,84,85]. Even with these examples, specific antiviral therapies or vaccines are not available to combat most orthoflavivirus infections, with IGUV being no exception. It is noteworthy that among mosquito-borne orthoflaviviruses, only a handful of recently licensed ones are available. These include DENV (tetravalent, live-attenuated dengue vaccine Dengvaxia^®^ manufactured by Sanofi Pasteur [86,87], and tetravalent dengue vaccine TAK-003 manufactured by Takeda Pharmaceuticals [88]), the YFV live-attenuated YF-VAX ^®^ 17D-204 manufactured by Sanofi Pasteur [89], the 17DD manufactured by Bio-Manguinhos/FIOCRUZ [90], and lastly the Japanese Encephalitis Virus (JEV), IXIARO^®^, a Vero cell-derived inactivated vaccine, manufactured by Valneva [91]. However, despite these notable exceptions, the majority of orthoflaviviruses lack specific antiviral therapies or vaccines, which pose significant challenges in managing their infections. But even though IGUV currently lacks any licensed vaccine or specific antiviral treatment, the fact that the virus appears to have a “low impact/low burden” currently, coupled with the fact that the traditional path of drug discovery is complex, time-consuming, and expensive, with a typical time required to bring a drug from concept to market usually exceeding a decade and costing billions of dollars [92,93], brings to light a probable negative outlook regarding the development of specific therapeutic approaches for IGUV, even though it is necessary and highly encouraged.

Much of the clinical management of patients infected with arboviruses aims only to alleviate the symptoms and complications associated with the infection. Symptomatic treatment focuses on relieving symptoms with analgesic, antipyretic, and non-steroidal anti-inflammatory drugs (NSAIDs), along with counseling the patient to stay adequately hydrated, especially if experiencing vomiting, diarrhea, or fever, as well as recommending proper rest. Additionally, regular medical monitoring is advised to monitor disease progression and potential complications, especially for at-risk groups such as pregnant women and the elderly. The lack of specific antivirals for IGUV represents a significant gap in the ability to deal with a potential IGUV emergence, leaving the medical and scientific community devoid of specific therapeutic options. However, in the face of a potentially devastating outbreak of IGUV, it is crucial to consider alternative treatment strategies. A promising approach would be to explore the potential of drugs that have shown efficacy against other viruses of the *Flaviviridae* family. An example is niclosamide, originally an FDA-approved anti-helminthic medication. Drug screening studies have found its effectiveness against various orthoflaviviruses in experimental animal models, including the Zika virus (ZIKV) [94,95], by inhibiting viral production and reducing inflammatory response [96]. Additionally, ribavirin, a synthetic nucleoside analog widely used in the treatment of hepatitis B and C, has been shown to suppress ZIKV replication in cells [97,98], although with varying results in animal studies [97,99]. Another possibility is emetine, an FDA-approved compound for the treatment of amoebiasis, which has shown broad-spectrum antiviral activity [100], including ZIKV [101]. These examples underscore the importance of exploring repurposing and existing FDA-approved drugs as potential treatment candidates against IGUV, offering a valuable strategy in situations where specific therapeutic options are limited. Moreover, it is important to highlight that these medications are not currently used for antiviral purposes or orthoflaviviruses infections.

To mitigate the risk of IGUV infection, it is imperative to implement general prevention measures, which are acknowledged as efficacious in averting other arboviruses. Key among these strategies are vector controls and the elimination of mosquito breeding sites, such as stagnant water containers, with the application of insecticides to diminish the adult mosquito populace [102,103]. Maintaining clean environments devoid of waste accumulation is pivotal in thwarting the proliferation of vectors of transmission. The use of insect repellents and clothing that covers most of the body, such as long pants and long-sleeved shirts, can mitigate skin exposure to mosquitoes while employing screens on doors and windows and sleeping under mosquito nets can provide supplementary protection indoors. The larvicide treatment of breeding habitats and aerial and truck spraying may also effectively reduce vector populations [104,105,106], and recently, the controlled release of *Aedes aegypti* mosquitoes carrying *Wolbachia bacterium* has been successful in reducing rates of arbovirus transmission [107,108,109,110]. However, determining the feasibility of this strategy for IGUV containment hinges on elucidating the true role of *Aedes* mosquitoes in the transmission of this disease.

Emphasizing the significance of community outreach and awareness of IGUV is also crucial for promoting preventive practices and reducing virus transmission. Public education can play a pivotal role in disseminating accurate information regarding the risks associated with IGUV, including its modes of transmission, symptoms, and preventive measures. By increasing awareness about IGUV, communities can be empowered to adopt behaviors that mitigate the risk of infection, as outlined previously. Past experiences with other orthoflaviviruses have demonstrated the significant benefits of public education in reducing the transmission of these diseases [111,112,113,114]. Critically, outreach and education can help combat misinformation and disinformation by promoting a simple yet accurate understanding of the disease and its consequences, thereby allowing for the early identification of initial outbreaks and consequently enabling the quicker implementation of control countermeasures. An added benefit of outreach efforts is empowering communities to make informed decisions in adopting behaviors that reduce the risk of infection, ultimately reducing the disease burden on public health systems and protecting public health.

To advance our understanding of IGUV beyond the development of effective countermeasures and diagnostic tools offering robust specificity and sensitivity, sustainable and coordinated efforts are required. These included comprehensive vector and host surveillance studies to identify the primary enzootic vectors and hosts of IGUV transmission. Although *Anopheles cruzii* has been suggested as a potential vector [51,63], its primatophilic feeding behavior raises questions about its role in IGUV transmission since there is strong evidence to suggest that birds may be the presumptive main enzootic host. Therefore, understanding the ecology and epidemiology of IGUV can contribute to our understanding of IGUV’s transmission dynamics and host range and, importantly, its potential for spillover and emergence into peridomestic and urban settings.

## 5. Conclusions and Future Perspectives

IGUV remains poorly characterized, with aspects of its transmission, ecology, epidemiology, and genetic diversity still not well understood. Currently, we lack a clear understanding of the actual burden of this virus in affected or at-risk areas. Additionally, there is a lack of rapid, accurate, and sensitive diagnostic tests suitable for implementation in hospitals or for use by clinicians in low-resource settings. Since IGUV circulates in regions of Brazil that are endemic for other arboviruses and febrile illnesses, accurate diagnosis could be challenging due to the similarity of its early symptoms with other illnesses. The enhancement of diagnostic capabilities will not only facilitate the early detection and treatment of IGUV infections but also contribute to a deeper understanding of its epidemiology and its dynamics of co-infection with other pathogens.

Although no documented human IGUV infections have been reported so far, we should not underestimate its potential emergence and impact on veterinary and human health. Urgent comprehensive epidemiological surveillance will require enhanced field and laboratory studies to identify the true breadth and depth of hosts and vectors of transmission, as well as understand the pathogenesis of IGUV infections in order to develop effective prevention and/or therapeutic countermeasures. Critically, there are no prevention methods specific to IGUV; however, already developed and effective protocols for well-known arboviruses can be readily deployed if a need arises. Current treatment is palliative since there is no antiviral therapy available, although the growing database of antivirals against orthoflaviviruses may offer effective repurposing options against IGUV infections. Lastly, the disruption of IGUV spillover and its emergence into peri-urban and urban habitats will likely benefit by leveraging our extensive experience and vast amounts of empirical data acquired when investigating similar pathogens to inform predictive models of emergence that have been successfully employed in recent years [115,116].

## Figures and Tables

**Table 1:** Documented circulation of IGUV among animals/arthropods.

Year	State	Positive/ Total	Species/Animal	Tests Performed	Ref
1989–1990	São Paulo	10/24	Ruddy ground dove (*Columbina talpacoti*)	HI	[42]
		2/3	Rufous-capped spinetail (*Synallaxis ruficapilla*)		
		1/1	Variable antshrike (*Thamnophilus caerulescens*)		
		1/2	Rufous gnateater (*Conopophaga lineata*)		
		1/3	Blue manakin (*Chiroxiphia caudata*)		
		4/7	White-bearded manakin (*Manacus Manacus*)		
		1/1	Swainson’s flycatcher (*Myiarchus swainsoni*)		
		1/2	Lesser elaenia (*Elaenia chiriquensis*)		
		3/6	Grey-hooded flycatcher (*Pipromorpha rufiventris*)		
		1/1	Southern rough-winged swallow (*Stelgidopteryx ruficollis*)		
		1/4	House wren (*Troglodytes aedon*)		
		2/3	Yellow-legged thrush (*Platycichla flavipes*)		
		1/4	Rufous-bellied thrush (*Turdus rufiventris*)		
		1/3	Yellowish pipit (*Anthus lutescens*)		
		2/17	House sparrow (*Passer domesticus*)		
		1/6	Red-eyed vireo (*Vireo olivaceus*)		
		5/5	Shiny cowbird (*Molothrus bonariensis*)		
		1/7	Masked yellowthroat (*Geothlypis aequinoctialis*)		
		1/2	Golden-crowned warbler (*Basileuterus culicivorus*)		
		1/1	Pectoral sparrow (*Tanagra pectoralis*)		
		2/7	Brazilian tanager (*Ramphocelus bresilius*)		
		2/14	Blue-black grassquit (*Volatinia jacarina*)		
		1/2	Sooty grassquit (*Tiaris fuliginosa*)		
		3/34	Double-collared seedeater (*Sporophila caerulescens*)		
		1/40	Rufous-collared sparrow (*Zonotrichia capensis*)		
1989	São Paulo	2/59	Wild Rodent	HI	[22]
1990		46/465	Wild Bird		
		1/8	Marsupial		
		1/13	Chicken		
		2/12	Duck		
		1/9	Teal		
1991		40/212	Wild Bird		
		1/5	Wild Rodent		
1992		58/296	Wild Bird		
		8/31	Wild Rodent		
		2/2	Bat		
1994	São Paulo	3 pools (90 mosquitoes)	*Anopholes cruzii*	Viral isolation, HI, PRNT and RT-qPCR	[51]
2004–2005	São Paulo	35	Equidae	HI	[52]
2007–2009	São Paulo	5			[53]
	Santa Catarina	7			
	Mato Grosso do Sul	21			
2009–2010	Mato Grosso do Sul	62		PRNT	[54]

Abbreviations: HI—hemagglutination inhibition test; PRNT—plaque reduction neutralization test; and RT-qPCR—quantitative reverse transcriptase–quantitative polymerase chain reaction.

## Data Availability

Not applicable.
